# AVATAR 2.0: next level communication systems for radiotherapy through face-to-face video, biofeedback, translation, and audiovisual immersion

**DOI:** 10.3389/fonc.2024.1405433

**Published:** 2024-10-08

**Authors:** Joseph B. Schulz, Laszlo Zalavari, Paulina Gutkin, Alice Jiang, Yi-Peng Wang, Clinton Gibson, Aaron Garza, Karl K. Bush, Lei Wang, Sarah Susan Donaldson, Billy W. Loo, Susan M. Hiniker, Lawrie Skinner

**Affiliations:** Department of Radiation Oncology, Stanford University School of Medicine, Stanford, CA, United States

**Keywords:** pediatric radiotherapy, anesthesia, video immersion, video distraction, patient communication, biofeedback, radio-transparent

## Abstract

**Purpose:**

This paper discusses an advanced version of our audiovisual-assisted therapeutic ambience in radiotherapy (AVATAR) radiolucent display systems designed for pediatric radiotherapy, enabling anesthesia-free treatments, video communication, and biofeedback. The scope of the AVATAR system is expanded here in two major ways: (i) through alternative mounting systems to accommodate a broader range of radiotherapy machines (specifically to fit robotic-arm and toroidal geometry photon radiotherapy and proton radiotherapy systems) and (ii) through additional hardware to provide video-calling, optimized audio for clear communication, and combined video inputs for biofeedback, translation, and other advanced functionalities.

**Methods and materials:**

Because robustness requires strong parts and radio-transparency requires thin, light parts, three-dimensional printing was used to rapidly prototype hollow structures and to iteratively improve robustness. Two system designs were made: one that mounts superior and another that mounts inferior to the patient’s head. Radiation dose measurements and calculations were conducted to assess dose perturbations at surface and depth due to the screen.

**Results:**

For 6-MV volumetric modulated arc therapy (VMAT) plans, with and without the screen, the mean and maximum dose differences inside the planning target volume were 0.2% and 2.6% of the 200 cGy prescription, respectively. For a single static beam through the screen, the maximum measured excess surface dose was 13.4 ± 0.5%, and the largest measured dose attenuation at 5-cm water-equivalent depth was 2.1 ± 0.2%. These percentages are relative to the dose without the screen at those locations.

**Conclusions:**

The radiolucent screen systems provided here are shown to give minimal dosimetric effects on megavoltage VMAT photon treatments. For static beams, however, surface dose effects should be considered when these beams pass through the thickest components of the screen. Design files are also provided.

## 1 Introduction

We previously developed an audiovisual system to assist radiotherapy patients relax without movement during treatment (1). The first versions of the system, named audiovisual-assisted therapeutic ambience in radiotherapy (AVATAR) were compatible with C-arm radiotherapy linear accelerators and some proton therapy machines. These radiolucent screens have now been used to successfully reduce anesthesia use, reduce patient anxiety, and reduce total treatment time in several hundred pediatric radiotherapy patients as part of a multi institution trial (1–4).

Cancer remains the largest cause of disease related mortality in children past infancy, and radiotherapy is key in treating 30%–50% of these cases (5, 6). Hence, a key driver of this work has been toward simplifying and improving pediatric radiotherapy treatments by replacing anesthesia with audiovisual immersion to keep these patients calm and still. The cost and logistics of combining radiotherapy and anesthesia are particularly pertinent to low- and middle-income countries where 80%–90% of childhood cancers occur (7). Acute toxicities from daily anesthesia include hypoxia, allergic reactions, and hyperthermia (8). In addition, infant animal studies have shown neuronal apoptosis from anesthesia (9, 10). Analysis on pediatric patient populations have found increased risk of learning disabilities, and long-term differences in language and cognitive functions with early exposure to anesthesia (11–13).

When considering an audiovisual system for radiotherapy, glasses, goggles, and screen-based systems are all viable options. While goggles, or glasses, can provide simple setup, they can create significant additional dose to the eye. The risk of cataract formation, for example, has been shown to be significant even at radiation doses less than 1 Gy (14). Digital goggles also suffer radiation damage and often exhibit display noise or failure when in the direct radiation beam. Devices in skin contact with the patient also need to be cleaned or replaced frequently. Projector-screen solutions allow the electronics to be outside of the treatment beam and away from the patient surface. This allows for interruption-free displays with minimal additional dose or attenuation from the presence of the thin screen. A downside is that projector screens can require more setup and alignment than glasses or goggles. For treatment sites outside of the head and neck, where direct radiation beams do not pass through the equipment, glasses or goggles may be more advantageous. Pediatric radiotherapy, however, predominantly involves the central nervous system.

In this paper, we have expanded the capabilities from a simple video display to a full two-way communication platform, adding face-to-face video calling, screen-sharing, translation, and biofeedback, all wirelessly to the patient inside the radiation treatment vault. New setup configurations developed also expand the scope of these systems to robotic arm radiotherapy (CyberKnife), and toroidal geometry machines, including tomotherapy, PET/CT, and Halcyon linacs. Three-dimensional (3D) printable designs are shared on GitHub (15). Dosimetric analysis of these screen-based systems on the patient dose for cranial sites is presented.

## 2 Materials and methods

To create clinically effective audiovisual systems, two critical design aspects are considered: (i) minimizing the screen’s impact on the radiation delivery and (ii) ensuring robustness for daily clinical use. Because most of the rigidity of an object comes from the outer shell (stiffness is proportional to the second moment of the cross-sectional area), we used 3D printing techniques to rapidly design and produce low-density, hollow parts.

### 2.1 Projector-screen design detail and evolution

During development, several screen materials were investigated. Paper was first considered for its lightness and poses little risk when collided with the gantry. However, paper is easily damaged both mechanically and by liquids. While lamination could be added for water repellency, shiny surfaces reduce image quality. The second material investigated was a 6-mm-thick foam board. This provided a rigid screen with a mass of 20 g that covered a 300-cm² area. Although light, the foam board did not sanitize easily nor last long mechanically. A combination of matte vinyl laminate and cardboard was found to be effective but with still limited mechanical toughness. Current AVATAR designs use a matte vinyl laminate (approximately 0.1 mm thick), backed with plastic or single ply carbon fiber reinforced plastic, that is another 0.2–0.3 mm thick. This screen is then supported by a hollow 3D printed plastic arm (4). The arrangement featured a matte, wipeable surface to enhance image display quality. The frame, weighing 10 g for the sheet and another 10 g for the support arm, ensured sufficient rigidity. This structure offered an average water equivalent thickness of 0.3–0.4 mm for the sheet and 1–2 mm for the support arm. To reduce the risk of collisions with the C-arm linac and to increase rigidity, a curved screen with a radius of approximately 0.6 m was selected. A flat screen alternative was also developed to provide a wider field of view for optical surface monitoring cameras. By far, the most common failure point was collision of the screen with the gantry or floor (when dropped).

Initial designs used carbon fiber sticks in between the 3D printed screen and 3D printed tube. The large mismatch between the rigidity and fracture toughness of the carbon fiber and 3D printed parts caused cracking. Better resilience was found with a slightly more pliable single-part 3D printed design. Extensive use also made apparent the need for a plastic frame at the top and sides of the screen to protect against impact damage.

Earlier designs relied on camera tripod links (dinkum links). These provided flexibility but led to slow and imperfect screen-projector alignment. Later designs removed the roll and yaw adjustments, ultimately utilizing only pitch, longitudinal, and vertical distance adjustments for simplicity and setup speed.

The wide projection angle of the commercially available battery-powered projectors dictated a screen projector distance of 0.3–0.6 m. Additional tele-converting lenses were tested. The majority of tested lenses provided unacceptable chromatic aberration and focusing artifacts. A relatively small condensation using a 1.4× tele-converting lens adapter provided acceptable image degradation. Detail on the current Cephalad mounting system is given in Section 2.4. Where space or access around the patient’s face is more critical, such as in the Cyberknife system, mirror glasses in conjunction with a caudad projector setup were developed (Section 2.5). The current versions of these designs are shared on GitHub (15).

### 2.2 Connection methods

**Figure 1:**
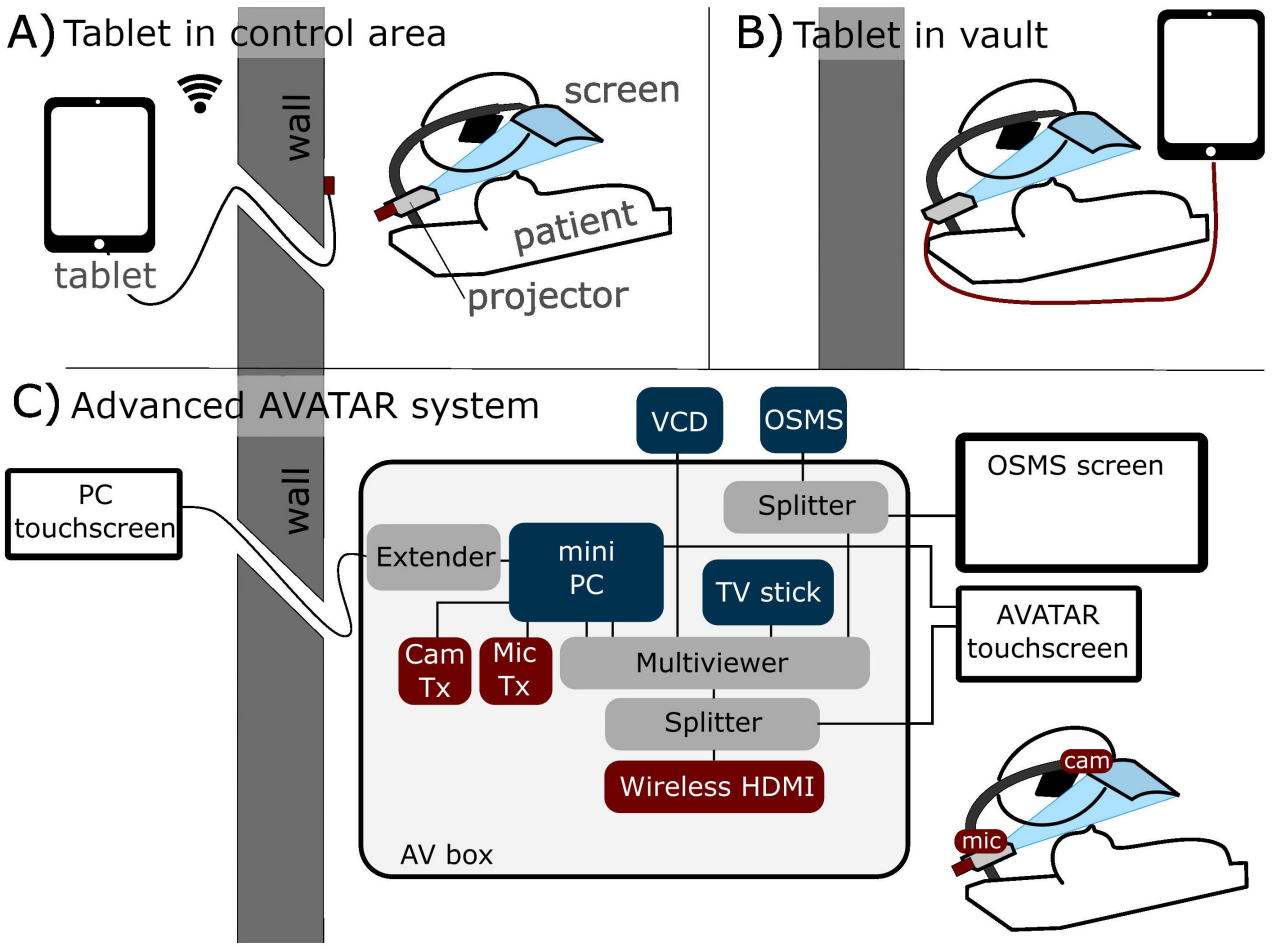
Schematic of three different connection methods. **(A)** Tablet-driven content from outside the treatment vault via wireless HDMI (allows control from outside). **(B)** Direct connection inside the treatment vault. **(C)** More advanced two-way communication system, with in-house AV box (audiovisual) that can send four inputs to the patient’s screen. This system also has touchscreens for setup both inside and outside the treatment vault.

The two main connection methods of the simple display system are illustrated in **Figures 1A** (wireless external tablet connection), and **Figure 1B** (direct connection). Connection method **Figure 1A** allows for control of the AVATAR display from outside the treatment vault. The wireless HDMI dongle is powered by the internal battery pack of the projector, so that the system can last several hours between charges. Connection method **Figures 1B** (direct connection) is the simplest and allows patients to be easily transitioned from watching on the tablet while waiting to watching on the treatment table. Drawbacks of method 1B are that the content cannot be controlled from outside. One variation on setup 1B is to use a USB-powered TV stick instead of the tablet. **Figure 1C** is a schematic of the connections and components of the more advanced two-way communication system. This uses a mini-computer and an HDMI Multiviewer to provide more advanced functionality, such as translating text and voice commands, webcam-communication, and integrating biofeedback systems. Further discussion is given in Section 2.3.

### 2.3 Two-way communication for video calling, screen-sharing, and patient feedback

A camera was positioned at the base of the screen, providing a view of the patient’s face. The microphone was placed further down the arm, superior to the head and outside of the radiation beam. These devices were connected to a minicomputer (GMKtec, Shenzen, China) to enable more interactive communication between the patient on the couch and family or staff outside the vault. Video conferencing software and its suite of features allows for intuitive screen sharing and remote control of the patient’s viewable media. Four main modes of operation are then provided with this setup. The camera, which is from first-person-view drone technology that combines a small camera and radiofrequency (RF) transmitter and an RF receiver (Skydroid UVC receiver, Shenzen, China) connected to the minicomputer. This allows wireless camera feed without having a PC on the treatment couch. The wireless microphone (Røde VideoMicro II and Wireless Go II, Røde, Sydney, Australia) uses a similar RF transmitter–receiver connection. To simplify content selection and the switching between modes of use a TV stick (Amazon fire TV stick, Amazon, Seattle, Washington, USA) was used to provide the content for audiovisual immersion. Noise suppression and voice clarity was amplified from the microphone using equalizer APO software (16) combined with a Werman, artificial intelligence (AI)–based noise suppression filter (17).

### 2.4 Cephalad design

**Figure 2:**
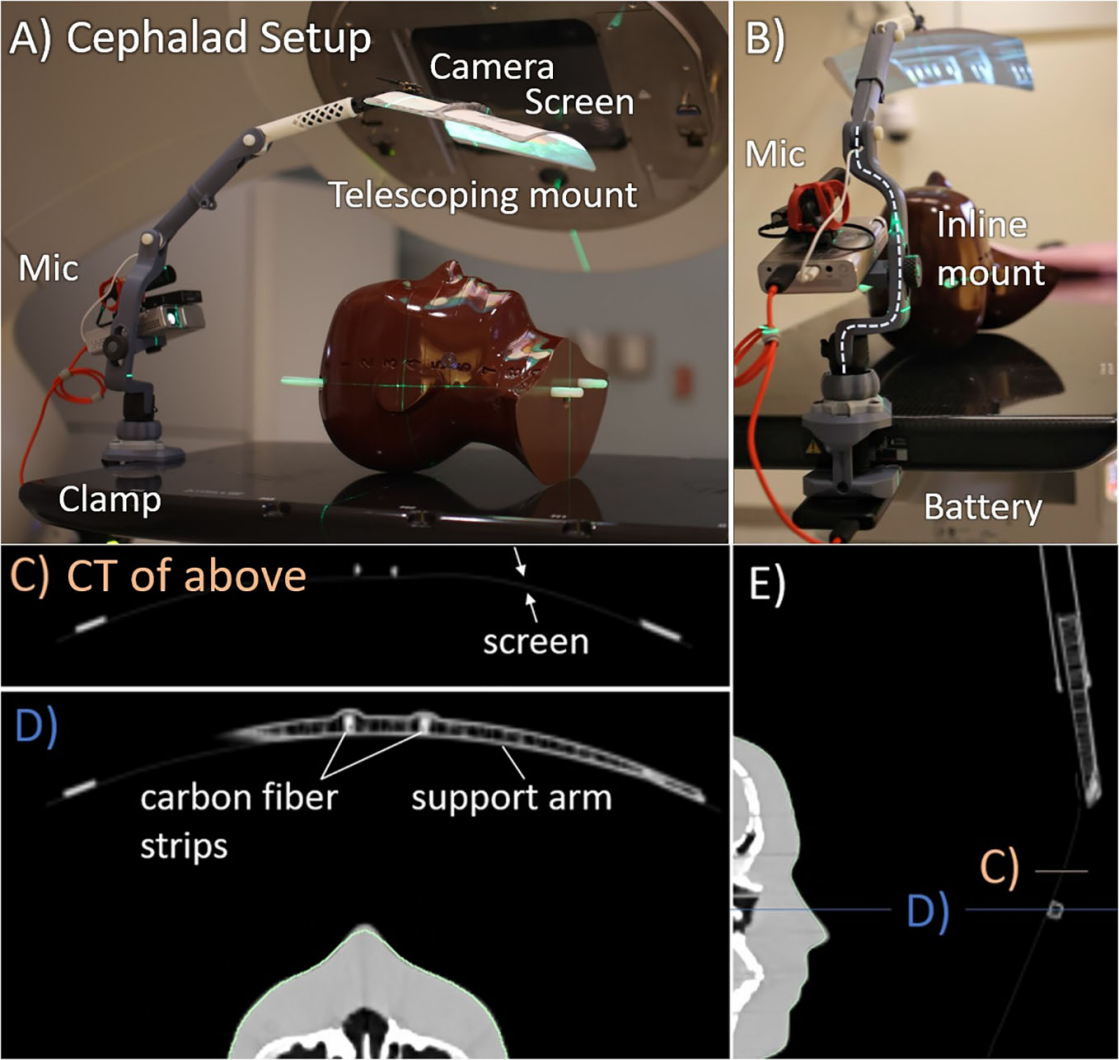
Cephalad AVATAR system setup and CT images. **(A)** Side view. **(B)** Superior view, looking down the treatment table. The computed tomography (CT) slices shown in **(C–E)** have Hounsfield unit (HU) range of −1,000 (black) to 400 HU (white). **(C, D)** Axial slices through the screen and hollow support arm. The support arm has wall thickness of 0.8 mm and infill fraction less than 10%. Similarly, the telescopic tube shown in **(E)** also has wall thickness of 0.8 mm and infill fractions of less than 10%.

The cephalad AVATAR system mounted to a portable projector at the head of the treatment table, which displayed video on the screen (**Figure 2**). This mounting point was chosen for C-arm linacs as it minimizes gantry collisions for axial arc treatments. To enable routine clinical use, a 3D printed telescoping arm design was developed. The screen was attached to the 3D printed arm by carbon fiber strips (**Figure 2D**), which provided sufficient flexibility and strength to withstand accidental collisions. Further adaptations included a louder speaker, a keystone correcting projector, and a small webcam. The screen is constructed from 0.25-mm-thick wipeable matte plastic sheet, which is supported by hollow 3D printed plastic arms that have an average water equivalent thickness of less than 2 mm. The system can be mounted at a single point.

### 2.5 Caudad design

To avoid mounting cephalad to the patient, which can impede some beam angles for cranial treatments, a rear projection based (caudad) system was developed. The wide throw angle of commercial projectors means that the projector to screen distance needs to be small to avoid excessive image size. When required, a 1.4× telephoto lens was employed to reduce the throw angle of the projector, which allowed a longer projection distance for a given image size. Longer focal length lenses or telephoto reducers were found to increase chromatic aberration and distortion and reduce the depth of the focal plane. For these reasons, designs that use very long projection distances were avoided.

**Figure 3:**
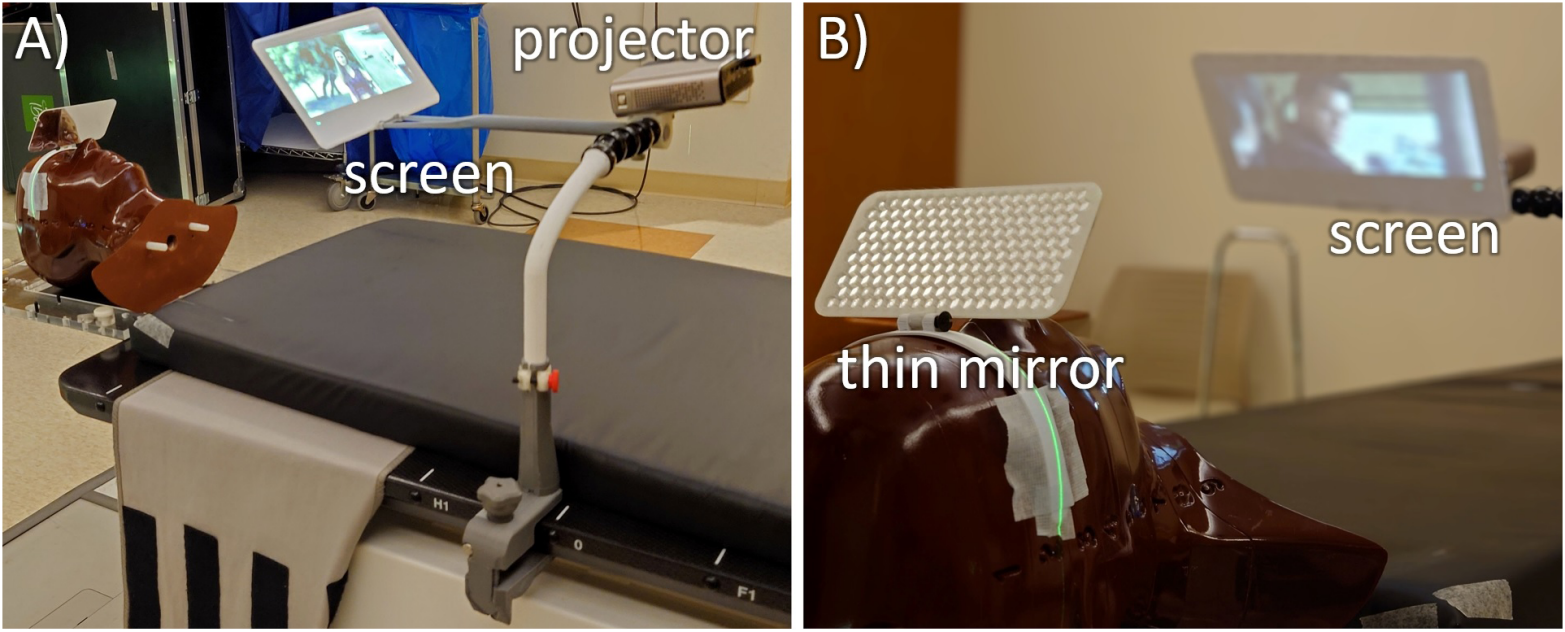
Two views of the mirror glasses with inferior-projection setup on a CyberKnife M6 robotic radiotherapy machine. **(A)** The system consists of 3D printed parts starting with C-clamps that connect to the treatment table (gray), a vertical telescopic arm (white) that connects to some dinkum links that provide setup flexibility. This holds an adjustable screen holding arm and the projector. **(B)** For optimal clearance around the head, this is all viewed through a thin plastic mirror.

The rear projection system developed in this work (**Figure 3**) consists of a vertical telescopic arm that mounts to the side of the patient. This has a mounting point for the projector and a small screen that extends in the superior direction. The single combined mount simplified setup and allowed for shorter projector screen distances which maximized image quality.

To keep a wide range of available beam angles and to minimize the possibility of collisions, the screen arm is kept well inferior of the patient’s head, and an aluminum laminated 14 cm × 7 cm mirror glass sheet of 2 mm thickness is attached to the patient’s mask that is adjusted to view the projector screen. Although this design requires a longer setup time due to the mirror glass adjustment, it adds the benefit of all electronics being placed far from the direct radiation fields and minimizes the size of the components around the patient’s head.

One use case of the caudal design is that of the CyberKnife^®^ (CK, Accuray, Madison, Wisconsin, USA) robotic radiotherapy system. Treatments on the system may be as long as 60-min duration, so implementing an AVATAR system for this can result in major improvements in patient experience. The reduced field size, the smaller SAD of 80 cm, and the non-coplanar beam angles pose a challenge for the cephalad design, increasing collision risk and direct beam incidence on the projector. These are overcome in the caudal design. The sensitive electronics are far from the target, and the beam entry rays can be assessed during treatment planning to select a mounting location for the AVATAR setup that prevents collision. The small mirror screen is close enough to the body that CK’s collision detection system will avoid it. If there are further concerns for collision remain, then any part of the AVATAR can be contoured in the treatment planning system (TPS) as part of the body, which will force the robot to avoid overlapping with that structure.

### 2.6 Dose calculations

**Figure 4:**
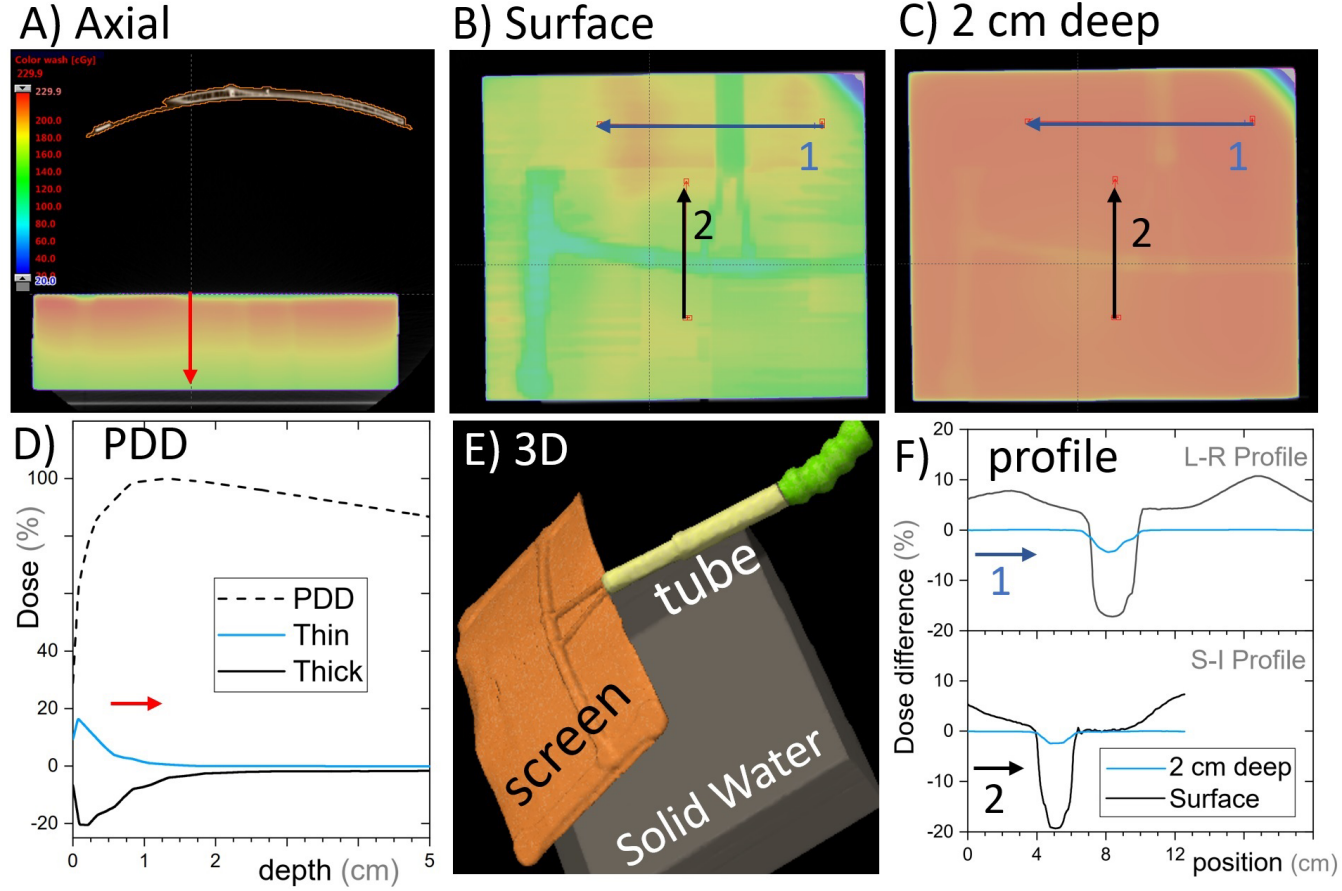
Single open anterior-posterior (AP) field calculated with and without the cephalad AVATAR system in place. **(A)** An axial slice of the AVATAR system and the solid water phantom. **(B)** Coronal slices at the surface of the phantom. **(C)** Coronal slices 2 cm deep in the phantom. The color wash is for the calculation with the screen present and is the same scale as given in **(A)**. **(D)** The depth dose profiles for the total (dashed line) and largest differential depth profiles (solid lines) that occur beneath thick and thin screen parts. **(E)** 3D-view to assist in understanding the setup orientation. **(F)** Differential dose profiles corresponding to the arrows in **(B, C)**, where differential is the calculated dose difference between with and without the screen, relative to the prescription dose. In Summary, the dose perturbation static beams on the screen is less than 5% at depths over 2cm. The surface dose perturbation, however, is larger.

The cephalad screen design was computed tomography (CT) scanned in two arrangements: (i) with a RANDO anthropomorphic phantom (Radiology Support Devices, Long Beach, CA) and (ii) a square water-equivalent plastic phantom. The latter allowed for ion chamber surface dose measurements, whereas the former allowed for a realistic treatment geometry. A simple treatment plan with an anterior-posterior (AP) beam was calculated on the solid water phantom with and without the AVATAR screen (**Figure 4**). The AP plan was 6 megavolt (MV), 200 monitor units (MU), with a 20 cm × 20 cm field, gantry, collimator, and couch angles at 0° with a source to phantom surface distance of 100 cm. The solid water phantom was 30 cm × 30 cm across with a vertical thickness of 7 cm for the surface measurement and 12 cm for the 5-cm-deep measurement. The dose was calculated using Acuros v15.6 dose calculation algorithm with a resolution of 1.25 mm within the Eclipse treatment planning system (v15.6, Varian Medical Systems, Palo Alto, CA, USA) using a standard beam model for a Varian TrueBeam linear accelerator.

**Figure 5:**
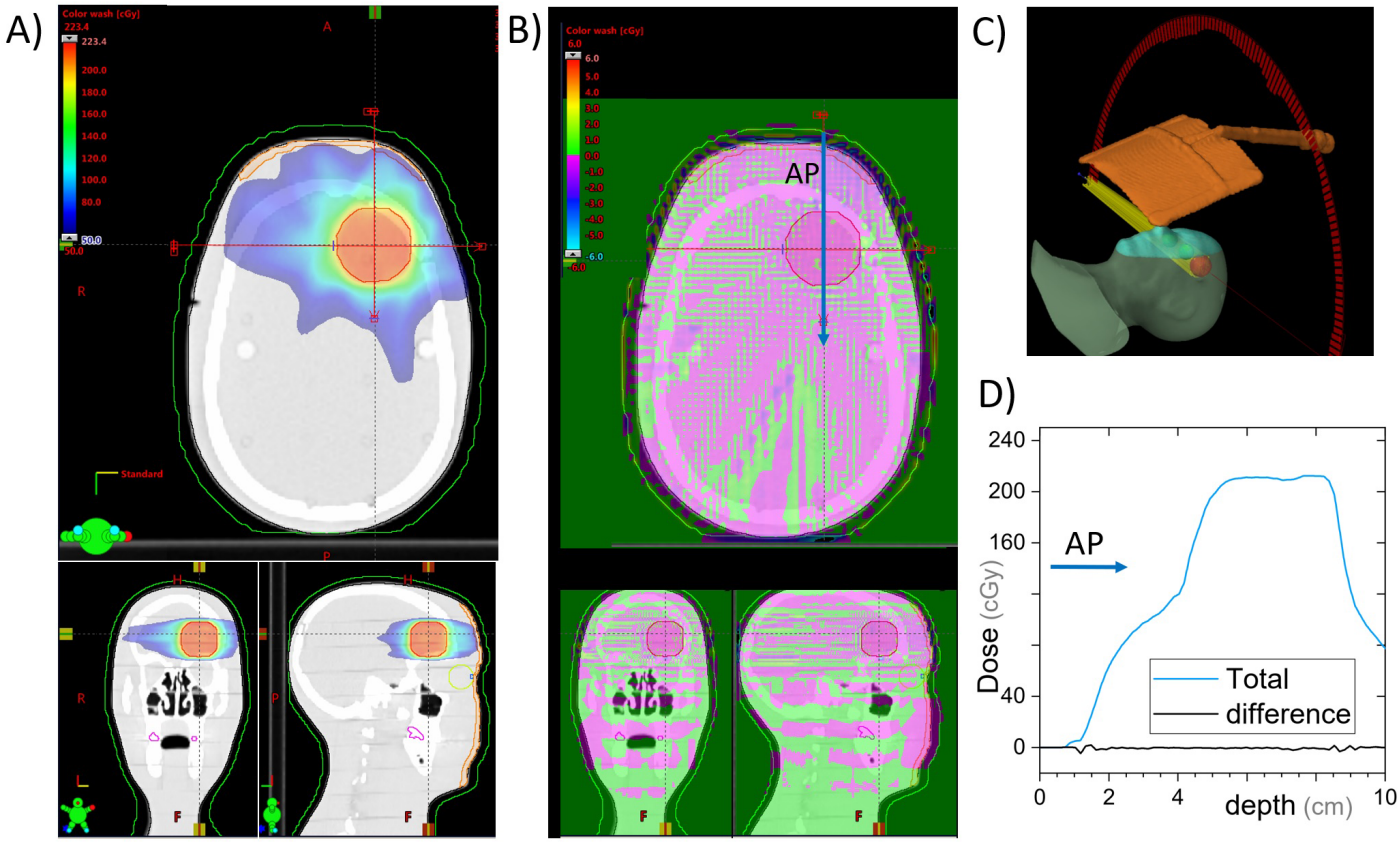
VMAT plan for a 3-cm-diameter spherical brain PTV. Dose is calculated with and without the cephalad AVATAR screen system. **(A)** Three views of the planned dose. **(B)** The differential dose between the plans with and without the AVATAR screen. **(C)** 3D view of the setup. **(D)** A beam profile in the anterior-posterior direction [at the location of the arrow in **(B)**]. The difference due to the presence of the screen is less than 3% of the prescription dose throughout and less than 0.5% in the vast majority of the body volume.

A more clinically realistic volumetric modulated arc therapy (VMAT) treatment plan was calculated on the RANDO phantom setup. The VMAT plan included a half-arc treatment with gantry angles from 90° to 270° (**Figure 5**). The plan was optimized on a spherical 3-cm-diameter planning target volume (PTV) placed in the brain superior and adjacent to the left eye. Optimization was performed using a ring structure expanded 1.5 cm from the PTV to maximize dose gradients outside the PTV, as is often done clinically. The prescription dose to this PTV was 200 cGy per fraction.

The caudal design was scanned in a single arrangement with the mirror glass attached to the same RANDO phantom as was used for the cephalad design but with a higher resolution (1-mm slice thickness) as dictated by our clinical workflow. Because static fields 3D plans are not clinically delivered with the CK system and entry through any structure can be dynamically avoided during treatment planning, the static beam configuration on the solid water phantom was not investigated for this design. A PTV of the same size and comparable location to the VMAT plan was delineated and prescribed a dose of 2,000 cGy in a single fraction to the 70 ± 5% isodose line as is common in stereotactic radiosurgery (SRS). The dose was calculated using Accuray’s Precision TPS and its in-built Ray-Tracing algorithm using the Iris collimator with 15-mm, 20-mm, and 25-mm field sizes and the VOLO optimizer. To assess the dosimetric changes due to the presence of the mirror glass, this structure was contoured and its mass and electronic densities were set to zero, and the plan was recalculated on the same CT scan.

### 2.7 Dose measurements

Dose with and without the cephalad version of the AVATAR screen (**Figure 2**) was measured using a Markus A10 parallel plate ion chamber (PTW dosimetry, Freiburg, Germany) placed in the solid water phantom in the same arrangement as the CT scan (**Figure 4**). The ion chamber measurements were made at two positions horizontally: (i) directly below the thickest part of the plastic support arm and (ii) underneath the 0.25-mm-thick plastic sheet a few cm offset from the support arm (**Figure 4**), each at depths of 0 cm and 5 cm, using a constant source-detector distance of 100 cm.

### 2.8 Cone beam computed tomography perturbations

**Figure 6:**
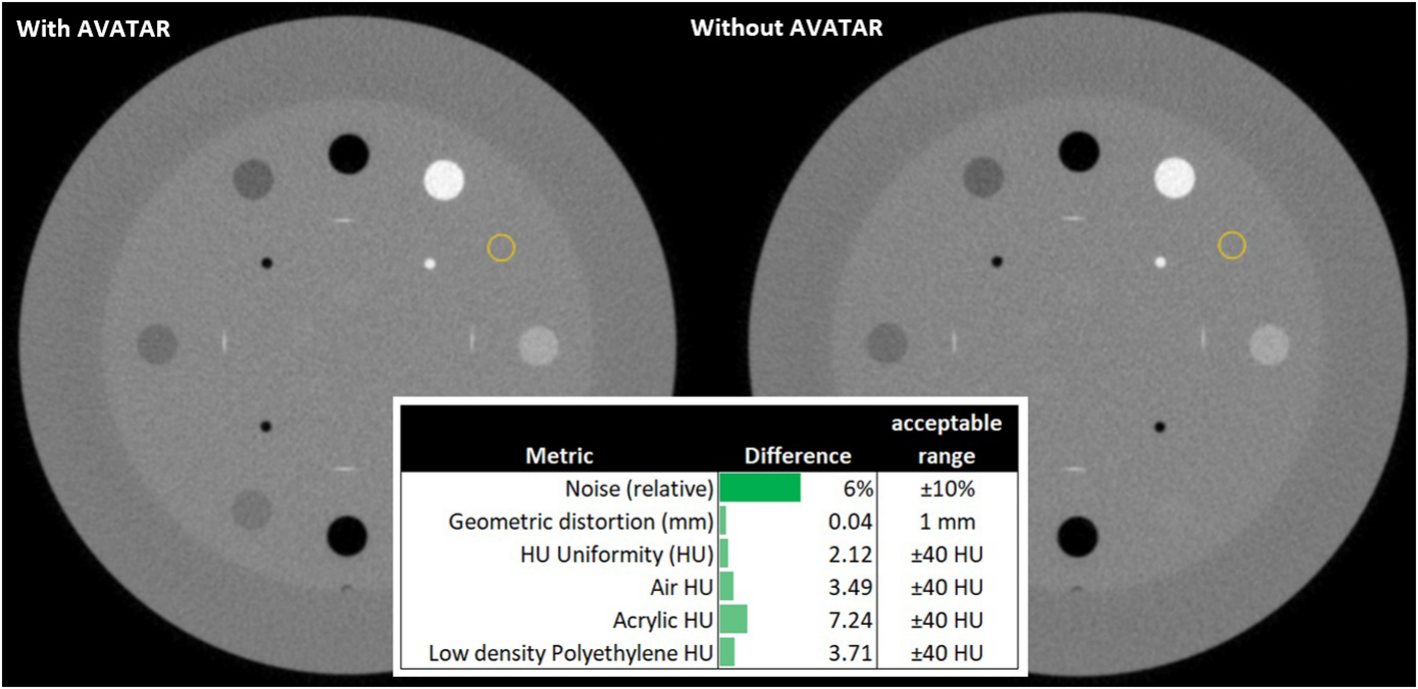
Slice from a cone beam computed tomography (CBCT) scan of a Catphan image quality phantom measured with and without the AVATAR screen in the beam immediately anterior of the phantom. “Head mode” on a Varian TrueBeam v3.0, 100 kVp, full-fan, 200° rotation, standard reconstruction. Differences shown are from subtracting results with and without the AVATAR screen in the beam. All deviations are well within the acceptable clinical tolerances. Visual inspection of the images shows no observable differences.

To investigate the effects of the AVATAR system on the on-treatment kV imaging systems, quality assurance measurements were made with and without the AVATAR screen in the kV cone beam computed tomography (CBCT) beam immediately anterior to a Catphan 504 cylindrical image quality phantom (The Phantom Laboratory, Salem NY, USA). These image quality tests were performed on a Varian TrueBeam OBI system running TrueBeam software version 3.0. A head mode CBCT was selected which had a kVp of 100, full-fan bow tie filter and a 200° rotation range. The standard reconstruction option was chosen. The results of this test are shown in **Figure 6**. Analysis was performed using SunCHECK v 4.2.1 (Sun Nuclear, Melbourne, Florida, USA).

## 3 Results

### 3.1 Dose calculation results

Dose calculations for the static AP beam direction are shown in **Figure 4**. This represents a worst-case scenario of a large field size irradiating the whole screen for the entirety of the beam on time. The general behavior was that the screen increases surface dose, and slightly reduces dose at depth (**Figure 5D**). The dose reduction at depth was more robust and was less than 2% everywhere for depths greater than 3 cm (where percentage is defined relative to the maximum dose on central the axis). The strongest attenuation was observed directly below the thickest parts of the screen. The excess surface dose, however, was seen as broad humps, that are more broadly spread across the phantom, consistent with electrons scattered from the screen (**Figure 4B**).

Clinically, only anatomic sites in the head or neck would have beams passing directly through the screen. In our clinic, whole-brain irradiation, and some larynx treatments are delivered with lateral beams, whereas the vast majority of the remaining head and neck sites such as oral cavity, neck, brain, ocular, brain stem, and cervical spine are treated using VMAT or conformal-arc techniques. The dose calculations and measurements for the AP beams were in agreement (Section 3.2), so the treatment planning system was used to calculate dose with and without the presence of the screen for a VMAT treatment on a phantom (Section 2.6). The calculated dose differences are given in **Figure 5**.

For the cephalad design calculations on the C-arm accelerators, the maximum dose difference due to the presence of the screen was 2.6% of the 200 cGy prescription, whereas the mean dose difference was 0.2% inside the PTV. Globally, the maximum dose difference was 6.2%. The eye lens structures on the phantom received a mean dose of 1.3 cGy with the screen and 1.2 cGy without.

**Figure 7:**
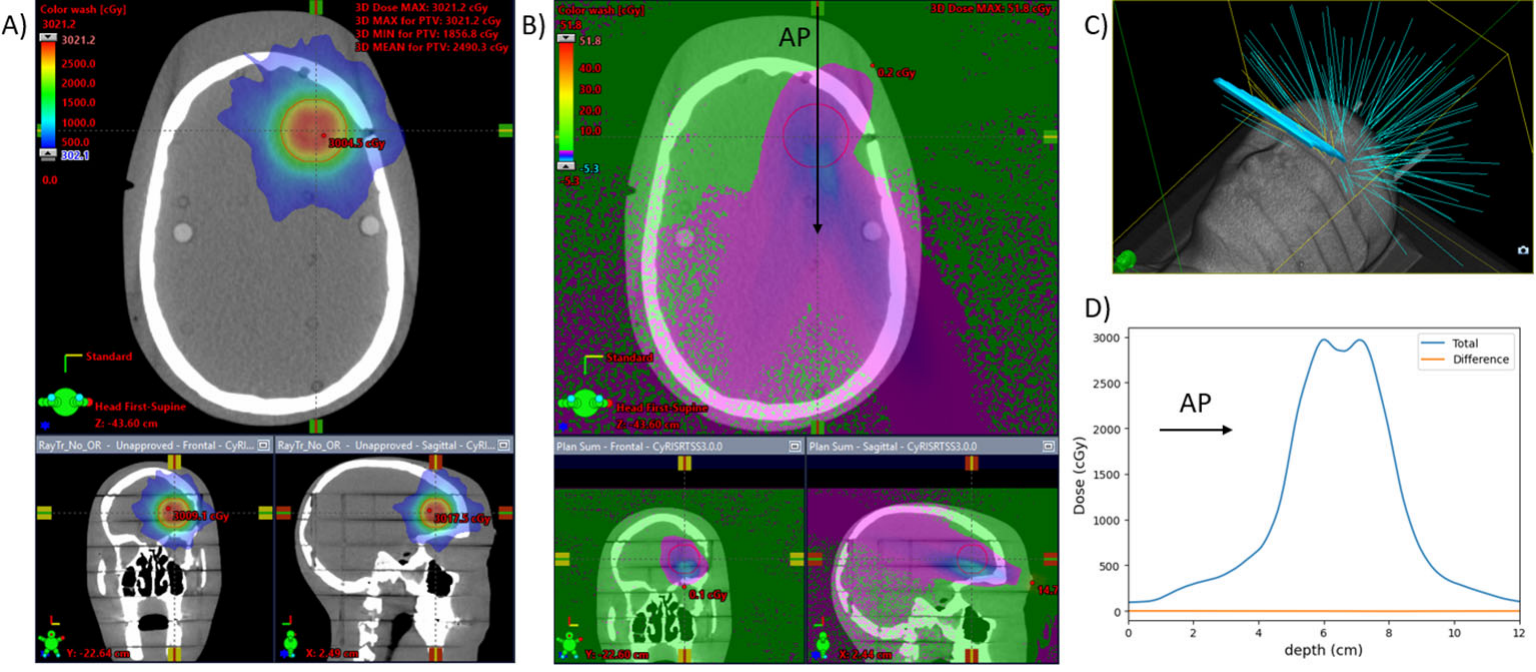
CyberKnife plan for a 3-cm-diameter spherical brain PTV. Dose is calculated with and without the mirror glass. **(A)** Three views of the planned dose with the mirror glass. **(B)** The differential dose between the plans with and without the mirror glass. **(C)** 3D view of the beams for the treatment. Eleven of the 121 beams are incident on the mirror glass structure, responsible for 8% (510) of the total MUs (6,380). **(D)** A beam profile in the anterior-posterior direction [at the location of the arrow in **(B)**]. The difference due to the presence of the screen is less than 0.2% of the prescription dose throughout.

Dose calculations for the caudal design on the CK system yielded a difference in the global and PTV maximum doses of 0.4 cGy (0.08% of the prescribed 2,000 cGy) and a difference of PTV mean dose of 2.2 cGy (0.11% of the prescription dose). Of additional interest, the maximum difference on dose to skin was 51.8 cGy, and the lenses received a mean dose of 43.0 cGy with the mirror screen present and 42.8 cGy without it. The calculated dose differences for this setup are found in **Figure 7**.

### 3.2 Measurement results

Ion chamber measurement results with the same setup as the calculations above are shown in **Table 1**. Excess surface dose percentages are defined as $100\cdot \frac{D_{S}-D_{N}}{D_{N}}$ , where *D_S_* and *D_N_* are the doses with and without the screen, were measured to be 10% to 15%, consistent with the dose calculations (**Figures 4B, F**). At 5 cm depth, the measured dose reductions were 2.1 ± 0.2% (through thickest part of the screen) and 0.0 ± 0.2% (through the thin sheet only). These percentages are relative to the dose without the screen at the respective locations (**Table 1**).

**Table 1:** Dose differences measured with an A10 parallel plate ion chamber due to presence of the AVATAR screen for a 6-MV anterior-posterior beam.

Screen part	Surface dose (% of Rx)	5-cm-depth dose (% of Rx)
Support arm	10.1 ± 0.5%	−2.1 ± 0.2%
Sheet, 0.25 mm	13.4 ± 0.5%	0.0 ± 0.2%

### 3.3 Two-way communication system testing results

**Figure 8:**
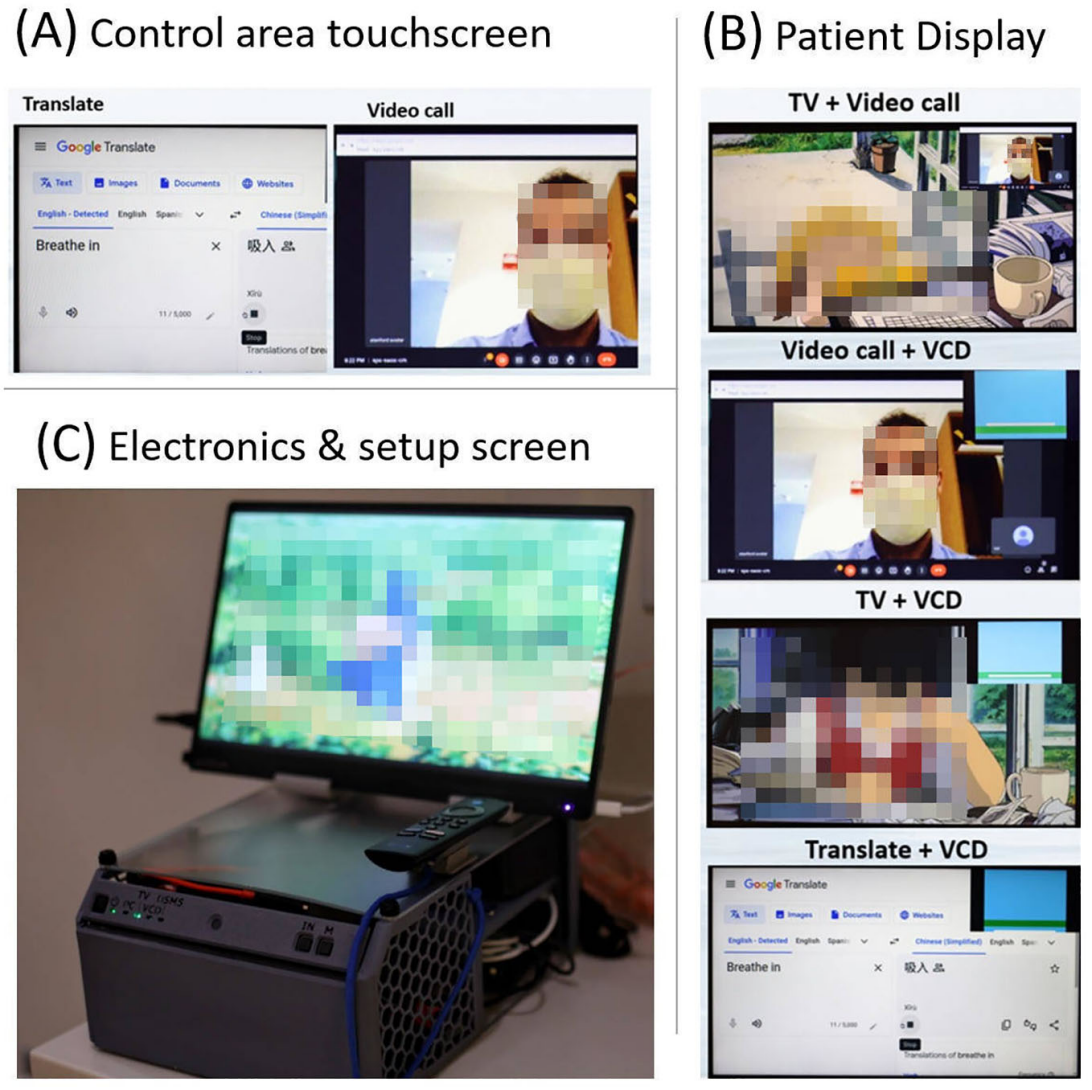
**(A)** Example screen shot from the control area touchscreen, **(B)** four example screenshots showing mixed mode usage from the patient display, and **(C)** the in-vault electronics and setup screen (mini PC, HDMI switch, kVM extender to control area screen, and TV stick) in a 3D printed enclosure. This setup screen is mirrored with the radiolucent patient display.

Testing of the audio and video signals showed significantly more clarity, for voice audio recordings than the in-vault wall microphone. With the wall mounted microphone, it is common in our clinic for us to not be able to comprehend the words spoken, whereas, with the AVATAR-mounted microphone, comprehension is trivial. This is presumed partly because of the AI-based noise suppression and partly because of the ~10× smaller distance between the audio source (patient’s head) and the microphone. **Figure 8** shows a selection of the content modes available with this system.

### 3.4 CBCT image quality perturbation results

**Figure 6** shows a slice of the CBCT scans obtained with and without the AVATAR screen immediately anterior of the Catphan 504 phantom. The differences in the SunCHECK analysis parameters for noise, geometric distortion, and HU values are given in the figure. All deviations are within clinical tolerances. The biggest deviation was the noise metric. We note, however, that this SunCHECK noise metric varies significantly (~5%) between sequential measurements even when no setup changes are made. Finally visual inspection reveals minimal observable differences between CBCT scans taken with and without the AVATAR screen in the beam path.

## 4 Discussion

### 4.1 Dosimetric commissioning

In this paper, we describe a novel system, developed in-house at Stanford University, designed to enhance the patient experience during radiotherapy. The minimal dose deviation of 0.2%, both with and without the screen, for the mean dose to the PTV in the VMAT plan aligns with the screen’s low water-equivalent thickness. It is important to note that the treatment beam is only interrupted by the screen for a portion of the treatment arc. In the Cephalad design with the 180° VMAT arc example, there is an additional dose of 0.1 cGy eye-lens dose (1.2 cGy vs. 1.3 cGy) for the 200-cGy PTV dose treatment. This additional dose would translate to less than 3 cGy for a full 6,000-cGy treatment course. While this is already a very small dose perturbation, the dose differences may be further reduced by utilizing full arcs (as opposed to the 180° arc studied) or by using avoidance sectors, in which case the screen can be completely avoided. However, we caution that, for VMAT arc therapy, avoidance sectors likely have a larger plan quality detriment than the ~0.2% mean dose deviation from the presence of the screen. In the context of static gantry AP fields, the surface dose was measured to increase up to 25% more than that without the screen. For example, an AP/PA plan would result in a maximum dose increase of 12.5% on the anterior surface of a patient in the supine position.

We note that the results reported here are lower than earlier reported dosimetry measurements (2), as the prior work was measured on the thicker, previous generation screen system, which had up to five times the water equivalent thickness of the currently used screens. The prior measurements were also made using optically stimulated luminescence dosimeters (nanoDots, LANDAUER, Glenwood, IL, USA), which are screened and certified to have a 5% uncertainty in absolute dose.

Dosimetry of the caudally mounted screen design was tested in the setting of SRS on the CK system where it is likely to see clinical usage. Dose calculations on an anthropomorphic phantom showed that the mean dose difference to a 3-cm-diameter spherical PTV was 0.11% of the prescribed 2,000-cGy single-fraction treatment between having the mirror glasses on, or off, of the phantom. The presence of this reflective sheet resulted in an additional dose of 0.2 cGy to the lenses.

For MeV electron beam therapy, kilovoltage therapy, or proton therapy, we advise against allowing the treatment beam to pass through the screen or its supporting structures. The rear-projection setup described in this work is provided such that the AVATAR system can be mounted in one of the two positions without disrupting such treatments. Given adequate quality assurance, the possibility of electron, proton, or heavy ion beams entering solely through the 0.3-mm-thick plastic sheet, and not through any of the support arms, may be reasonable.

### 4.2 Mechanical and human failure modes

From the experience designing, developing, and manufacturing 20–30 systems, coupled with multiple years of iterative design of resin 3D printed parts, a common trend was observed: these parts tended to crack over time. As a result, their application has been restricted to components with lower requirement for mechanical impact. Likewise, approximately 5 out of the 50 dinkum links demonstrated cracks after extended periods of use. The cracks were presumed to result from a combination of radiation damage and the strain associated with clinical use. The dinkum links were removed from later designs to improve reliability.

To minimize disruption of video viewing, pediatric patients were provided with a tablet while they were waiting. Once brought to the treatment vault, the video display was transitioned to the AVATAR projector screen system. This workflow was found to help patient relaxation and improve anesthesia-free compliance. Prior to treatment, child life service staff also assisted by introducing the system setup to the patient and facilitated a selection from a choice of media.

## 5 Conclusions

The implementation of the AVATAR system at Stanford and other radiotherapy centers has substantially decreased anesthesia usage, treatment durations, and overall treatment cost (1, 4). The 3D printable designs provided in this work enable interested parties to use and further develop this communication platform to reduce the daily anesthesia burden and generally improve pediatric radiotherapy. The advanced functionalities of video calling, translation, and respiratory gating biofeedback greatly expand the utility of these radiolucent screens in radiotherapy for improved patient experience and increased treatment efficiency. Future directions include further advancing the software toward providing positioning hints and beam status information to the patient, as well as monitoring additional biomarkers such as heart rate and respiration amplitude and frequency.

## Data Availability

The datasets presented in this study can be found in online repositories. The names of the repository/repositories and accession number(s) can be found in the article/supplementary material.
